# 

**Published:** 1877-05

**Authors:** 


### AMERICAN MEDICAL ASSOCIATION.

PHILADELPHIA, 1400 PINE St., S. W. Cor. Broad.

The Twenty-eighth Annual Session will be held in the city of Chicago, Ill., on Tuesday, June 5, 1877, in Farwell Hall, at 11, A. M.

"The delegates shall receive their appointment from permanently organized State Medical Societies, and such County and and District Medical Societies as are recognized by *representation in their respective State Societies*, and from the Medical Department of the Army and Navy of the United States."

"Each State, County, and District Medical Society entitled to representation, shall have the privilege of sending to the Association one delegate for every ten of its regular resident members, and one for every additional fraction of more than half that number: *Provided*, however, that the number of delegates for any particular State, territory, county, city, or town shall not exceed the ratio of one in ten of the resident physicians who may have signed the Code of Ethics of the Association."

Secretaries of Medical Societies as above designated are earnestly requested to forward, *at once*, lists of their delegates. Will you kindly send to the undersigned a list of your members with their residences, in order that a correct record may be made of all who are in affiliation with this body?

SECTIONS.

"The Chairmen of the several sections shall prepare and read in the general sessions of the Association, papers on the advances and discoveries of the past year in the branches of science included in their respective Sections. * * * *"—By-Laws, Art. II., Sec. 4.

Practice of Medicine, Materia Medica, and Physiology: Dr. P. G. Robinson, St. Louis, Mo., Chairman; Dr. B. A. Vaughan, Columbus, Miss., Secretary.

Committee appointed to report to this Section—on Clinical Observations: Dr. N. S. Davis, Ill., Chairman; Dr. H. A. Johnson, Ill.; Dr. J. B. Johnson, Mo.

Obstetrics and Diseases of Women and Children: Dr. James P. White, Buffalo, N. Y., Chairman; Dr. Robert Battey, Atlanta, Ga., Secretary.

Surgery and Anatomy: Dr. ———, Chairman; Dr. Moses Gunn, Chicago, Ill., Secretary.

Medical Jurisprudence, Chemistry, and Psychology: Dr. Eugene Grissom, Raleigh, N. C., Chairman; Dr. E. A. Hildreth, Wheeling, W. Va., Secretary.

State Medicine and Public Hygiene: Dr. Ezra M. Hunt, Metuchen, N. J., Chairman; Dr. D. R. Wallace, Waco, Texas, Secretary.

"Papers appropriate to the several Sections, in order to secure consideration and action, must be sent to the Secretary of the appropriate Section at least one month before the meeting which is to act upon them. It shall be the duty of the Secretary to whom such papers are sent, to examine them with care, and, with the advice of the Chairman of his Section, to determine the time and order of their presentation, and give due notice of the same. * * * *"—By-Laws, Art. II., Sec. 5.

The following Committees are expected to report:

On Influence of Climate on Pulmonary Diseases in Florida: Dr. E. T. Sabal, Fla., Chairman.

On Animal Vaccination: Dr. Henry A. Martin, Mass., Chairman.

On the Inheritance of Syphilis: Dr. J. W. Thompson, Ky., Chairman.

On Prize Essays: Dr. N. S. Davis, Ill., Chairman.

On Necrology: Dr. S. C. Chew, Md., Chairman.

On Catalogue of National Library: Dr. H. C. Wood, Pa., Chairman.

WM. B. ATKINSON,
*Permanent Secretary.*

### BOOKS AND PAMPHLETS RECEIVED.

The Practitioner's Hand-book of Treatment; or The Principles of Therapeutics. By J. Milner Fothergill, M. D., etc.

The Microscopist; A Manual of Microscopy and Compendium of the Microscopic Sciences, etc., etc. Third Edition, rewritten and greatly enlarged. By J. H. Wythe, A. M., M. D.—1877.

A Course of Practical Histology; being an introduction to the use of the microscope. By Edward Albert Schäfer, Asst. Prof. Physiology in University College, London, 1877.

Principles of Theoretical Chemistry, with special reference to the Constitution of Chemical Compounds. By Ira Remsen, M. D., Ph D., etc., 1877.

The Tonic Treatment of Syphilis. By E. L. Keyes, A. M. M. D., etc., 1877.

Annual Report of the Supervising Surgeon-General of the Marine Hospital Service of the U. S., for 1875. John M. Woodworth, M. D. 1876.

A Practical Treatise on the Diseases of Children. By J. Forseith Meigs, M. D., and William Pepper, A. M., M. D., Sixth Edition, Revised and Enlarged. Philadelphia. Lindsay & Blackiston, 1877. Frice $6.00.

Myelitis of the Anterior Horns or Spinal Paralysis of the Adult and Child, by E. C. Seguin, M.D. New York, G. P. Putnam's Sons, 1877. Price $1.50.

General Index to the New York Medical Journal, from April, 1865, to June, 1876. By James B. Hunter, M. D., New York. Appleton & Co., 1877.

Report of the Board of Health of the State of Georgia for 1876, with appendix, etc.

Transactions of the Nebraska State Med. Society at its 6th, 7th and 8th Annual Meetings.

Annual of Syracuse University. 1876-1877.

An Essay on New South Wales, the Mother Colony of the Australias. By G. H. Reed. 1876.

Principia; or, Basis of Social Science. By R. J. Wright.

Illuminating Oils in Michigan. A Lecture. By R. C. Kedzie, Member of the Board of Health.

On Some Conditions, Physical and Rational, in Effusions of the Pleura. By Beverly Robinson, M. D.

Annual Announcement of the Medical College of the Pacific. Session of 1877.

Annual Reports of Cook County Agent, Warden Insane Asylum and Poor-house, Medical Superintendent Cook County Insane Asylum, Warden Cook County Hospital, Cook County Physician, and Coroner, for the year 1876.

Catalogue of the Trustees, Professors and Students of the Jefferson Medical College for Session 1876-7.

First Annual Report of the State Board of Health of Wisconsin—for 1876.

On the Differential Indications for the Use of the Faradic and Galvanic Currents. By A. D. Rockwell, M. D.

Considerations in Relation to Diseases of the Joints. By David Prince, M. D.

The Operation of Vesico-Vaginal Fistula: A Comparison of the Methods of Operation of N. Bozeman, M. D., of New York, and Prof. Gustav Simon, of Heidleberg. Translated by A. C. Bernays, M. D.

Fifth Annual Report of the Board of Trustees of the New York Ear Dispensary. 1876.

A Case of Ovariotomy. By David Prince, M. D.

### ANNOUNCEMENTS FOR THE MONTH.

**MONDAYS.** SOCIETIES.
*Mondays, May 7 and* 21.—Chicago Medical Society. Regular meetings.
*Mondays, May* 14 *and* 28.—Chicago Soc. of Phys. and Surgeons. Regular meetings.
CLINICS. *Every Monday.*
At Eye and Ear Infirmary, 2 P. M.—Prof. Holmes.
At Central Dispensary (Wood and Harrison sts).—2 P. M. *Gynecological*, Dr. Adolphus; 3 P. M. *Diseases of Children* Dr. R. S. Hall.
At Mercy Hospital—2½ P. M.. *Surgical*, Prof. Andrews.
At Rush College--2¼ P. M., *Medical*, Dr. Bridge.
LECTURES. *Every Monday.*
At Rush College (Harrison and Wood sts.)—9 to 1 o'clock, Drs. Wadsworth, Jackson, Danforth and Knox. At Chicago College—8 to 11, Profs. Hatfield, Jewell, and Curtis; 3½, Quine.
At Woman's College—3 to 5, Profs. Paoli and Byford.

**TUESDAYS.** SOCIETIES.
*Tuesday, May* 8.—Academy of Sciences. Regular meeting at 8 P. M. (263 Wabash av.)
CLINICS. *Every Tuesday.*
At Eye and Ear Infirmary—2 P. M, ,Prof. Jones.
At County Hospital—2 P. M., *Medical*. Prof. Lyman. At 3 P. M., *Surgical*, Prof. Bogue.
At Mercy Hospital, 2 P. M., *Medical*, Prof. Hollister.
LECTURES. *Every Tuesday.*
At Rush College—9 to 1, Drs. Owens, Bridge, Strong and Case. At Chicago College—9 to 11, Profs. Jewell and Byford.
At Woman's College—9 to 11, Profs. Hayes and Earle.

**WEDNESDAY.** CLINICS. *Every Wednesday.*
At County Hospital—2 P. M., *Ophthalmological*, Dr. Montgomery; 3 P. M., *Gynecological*, Prof. Fitch.
At Mercy Hospital—2½ P. M., *Ophthalmological*, Prof. Jones.
At Central Dispensary—2 P. M., *Surgery*, Dr. Loomis; *Diseases of Chest*, Dr. Ingals; 3, *Gynecological*. Prof. Etheridge.
LECTURES. *Every Wednesday.*
At Rush College—9 to 1, Drs. Wadsworth, Ingals and Sawyer. At Chicago College—9 to 11, Pro s. Hyde and Steele; 3½, Jones.
At Woman's College—9 to 11, Prof. Marguerat and Dr. Hale.

**THURSDAYS.** CLINICS *Every Thursday.*
At Eye and Ear Infirmary—2 P. M., Prof. Hotz.
At Mercy Hospital—2½ P. M., *Medical*, Prof. Davis.
At Rush College—2 P. M., *Medical*, Prof. Ross; 3 P. M., *Diseases of the Nervous System*, Prof. Lyman.
At Central Dispensary—2 P. M., *Surgica'*, Dr. Graham.
LECTURES. *Every Thursday.*
At Rush College—9 to 1. Drs. Hayes, Bridge, Strong and Case. At Chicago College—9 to 11, Prof. Quine and Steele; 3½, Byford and Roler.
At Woman's College—9 to 11, Profs. Thompson and Graham.

**FRIDAYS.** SOCIETIES.
*Friday, May* 11.—State Microscopical Society of Illinois. Regular meeting, 8 P. M.
CLINICS. *Every Friday.*
At County Hospital—2 P. M., *Medical*, Prof. Lyman; 3 P. M., *Surgical*, Prof. Bogue.
At Mercy Hospital—2½ P. M., *Medical*, Prof. Nelson.
At Central Dispensary—2 P. M., *Diseases of Chest*, Dr. Harroun; 3 P. M., *Dermatological*, Dr. Maynard.
LECTURES. *Every Friday.*
At Rush College—9 to 1, Drs. Wadsworth, Jackson, Strong and Knox. At Chicago. College—8 to 11, Profs. Jones, Hyde, and Curtis; 3½, Hatfield.
At Woman's College—9 to 11, Profs. Bartlett and Blake.

**SATURDAYS.** CLINICS. *Every Saturday.*
At Chicago College—1½ P. M., *Surgical*, Prof. Andrews or Isham; *Gynecological*, Prof. N lson; 2½ P. M., *Medical*, Prof. Johnson.
At Rush College—2 P. M., *Surgicl*, Prof. Gunn.
LECTURES. *Every Saturday.*
At Rush Colle e—9 to 1, Drs. Owens, Bridge, Sawyer and Danforth. At Chicago. College—8 to 11, Profs. Merriman, Quine and Byford; 3½, Hyde.
At Woman's College—9 to 11, Dr. Engert.

At all the above named Clinics visiting regular practitioners are, we believe, admitted.

At the South Side Dispensary (Chicago College) there are six daily special Clinics, for sections of the classes of the Chicago College.

